# Estimating active layer thickness and volumetric water content from ground penetrating radar measurements in Barrow, Alaska

**DOI:** 10.1002/gdj3.49

**Published:** 2018-01-09

**Authors:** E. E. Jafarov, A. D. Parsekian, K. Schaefer, L. Liu, A. C. Chen, S. K. Panda, T. Zhang

**Affiliations:** ^1^ Computational Earth Sciences Los Alamos National Laboratory Los Alamos NM USA; ^2^ Institute of Arctic and Alpine Research University of Colorado at Boulder Boulder CO USA; ^3^ Geology and Geophysics Department University of Wyoming Laramie WY USA; ^4^ Department of Civil & Architectural Engineering University of Wyoming Laramie WY USA; ^5^ National Snow and Ice Data Center Cooperative Institute for Research in Environmental Sciences University of Colorado at Boulder Boulder CO USA; ^6^ Earth System Science Programme Faculty of Science The Chinese University of Hong Kong Hong Kong China; ^7^ Department of Geophysics Stanford University Stanford CA USA; ^8^ Geophysical Institute University of Alaska Fairbanks AK USA; ^9^ College of Earth and Environmental Sciences Lanzhou University Lanzhou China

**Keywords:** GPR, ALT, VWC, permafrost, Barrow

## Abstract

Ground penetrating radar (GPR) has emerged as an effective tool for estimating active layer thickness (ALT) and volumetric water content (VWC) within the active layer. In August 2013, we conducted a series of GPR and probing surveys using a 500 MHz antenna and metallic probe around Barrow, Alaska. We collected about 15 km of GPR data and 1.5 km of probing data. Here, we describe the GPR data processing workflow from raw GPR data to the estimated ALT and VWC. We include the corresponding uncertainties for each measured and estimated parameter. The estimated average GPR‐derived ALT was 41 cm, with a standard deviation of 9 cm. The average probed ALT was 40 cm, with a standard deviation of 12 cm. The average GPR‐derived VWC was 0.65, with a standard deviation of 0.14.

## Dataset

Identifier: https://doi.org/10.3334/ornldaac/1355

Creator: Jafarov, E., A. Parsekian, K. Schaefer, L. Liu, A. C. Chen, S. K. Panda and T. Zhang

Title: Pre‐ABoVE: Active Layer Thickness and Soil Water Content, Barrow, Alaska, 2013

Publisher: ORNL DAAC

Publication year: 2016

Resource type: Dataset Version: 1.0

## Introduction

Active layer thickness (ALT) is the maximum depth of thaw of the surface soils in permafrost‐affected soils. ALT is identified as one of the essential variables for monitoring the status of permafrost (Brown *et al*., 2008). Ground penetrating radar (GPR) is a non‐invasive geophysical technology that allows nearly continuous spatial estimates of ALT and enables better understanding of permafrost distribution and thermal dynamics. GPR uses pulses of radio‐frequency (~25 MHz to 2.5 GHz) electromagnetic waves emitted at the ground surface to investigate the subsurface. The energy from these pulses reflects at boundaries between materials of contrasting dielectric permittivity (Neal, 2004; Jol, 2009). For each pulse, a receiving antenna at the surface records the transmitted and reflected waves, resulting in a record of received amplitude as a function of time. Thawed soil in the active layer has higher dielectric permittivity (~30 to 40) than frozen soils in permafrost (3–10), resulting in a strong reflection at the permafrost table and making GPR a useful tool to measure ALT.

Previous studies used GPR to measure ALT on relatively small scales from tens of metres (Arcone *et al*., 1998; Munroe *et al*., 2007) up to several 100 m (Hubbard *et al*., 2013; Gusmeroli *et al*., 2015). During our work in Barrow, Alaska, in August 2013, we collected a total of nearly 15 km of spatially continuous GPR ALT measurements. In addition to the GPR data, we collected 1.5 km of high‐density probe surveys (100 ALT probe measurements made per 100 m of transect length) and other sporadic mechanical probe surveys. The purpose of these GPR surveys was to validate the remotely sensed Active Layer Thickness (ReSALT) product (Schaefer *et al*., 2015). Here, we provide detailed descriptions and some insights on the methods we used to collect and process GPR data. Raw and processed datasets along with ALT probe data are available for download from the Oak Ridge National Laboratory (ORNL) Distributed Active Archive Center (DAAC). The processed datasets include all collected data from the GPR unit and high‐density data (Jafarov *et al*., 2016). The expanded header description for processed datasets can be found in Appendix A.

## 1 Site descriptions

**Figure 1:**
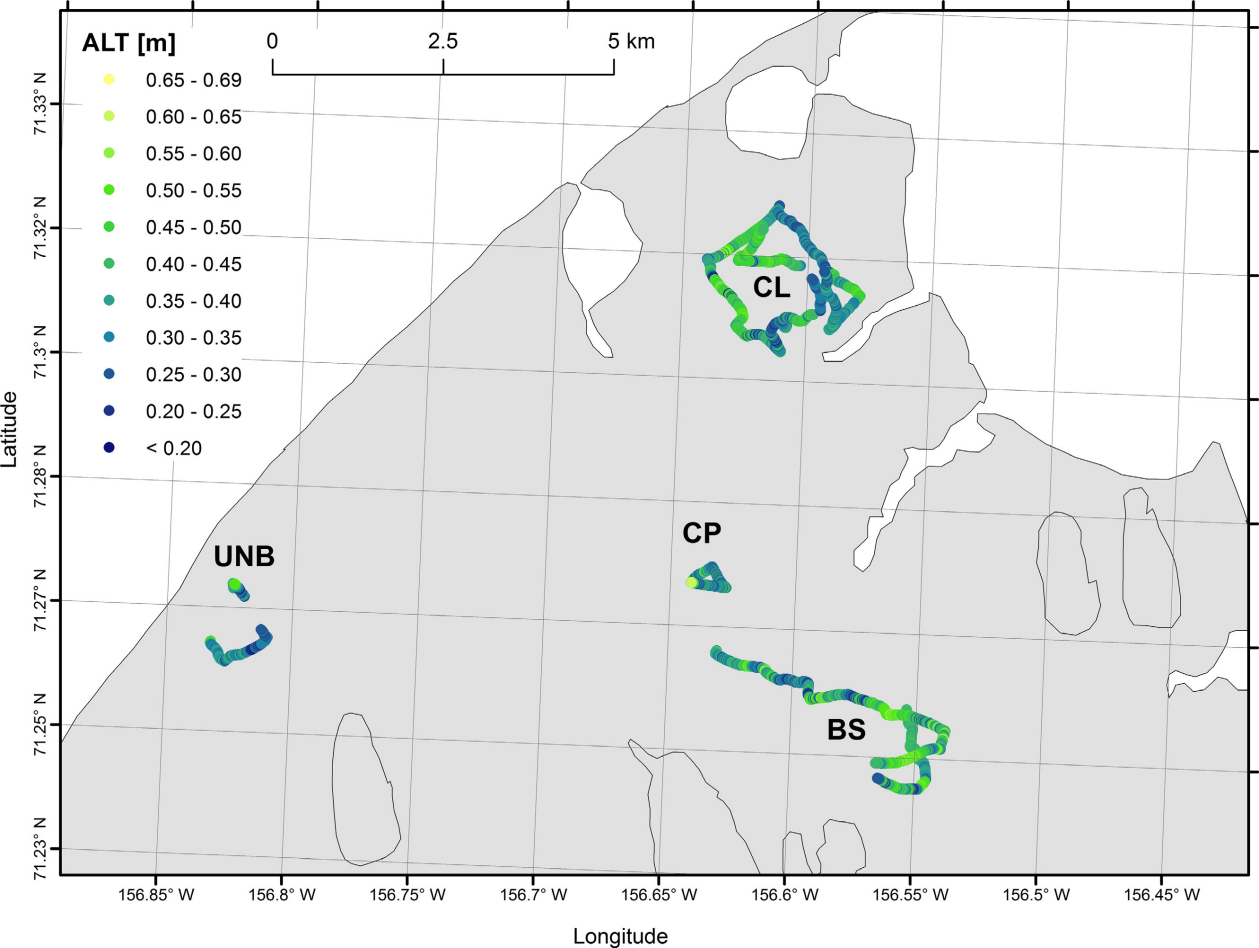
The ground penetrating radar tracks estimated active layer thickness for four sites at Barrow, Alaska, 2013.

We selected four sites for GPR ALT data collection, representing a range of environmental conditions commonly observed in the Barrow area (Figure 1 and Table 1). We named the four sites as following: Big Spot (BS), Circumpolar Active Layer Monitoring (CALM) site as CL, Central Plain (CP), and Upper Nunavak River Bay (UNB). Here, we briefly summarize the environmental conditions at each site. More detailed information on sites specifics and corresponding maps are given in Schaefer *et al*. (2015). The BS site consists of a series of drained thermokarst lake basins (DTLB) of varying sizes. The CL site consists of two large DTLBs separated by a narrow strip of upland tundra (Shiklomanov *et al*., 2010). The CP site is a matrix of high‐centre polygons with fully saturated soil and standing water over the ice wedges. The UNB site covers the upper portion of the Nunavak drainage basin and consists of undisturbed tundra surrounded on three sides by the Nunavak River and its tributaries. All four sites represent a mix of saturated and unsaturated soil conditions with undisturbed tundra conditions (CP), drained soils (UNB), and DTLB conditions (BS and CL).

**Table 1:** Four selected sites including corresponding codes, coordinates, and short description

Number	Site	Code	Latitude (deg)	Longitude (deg)	Selection criteria
1	Big Spot	BS	71.252975	−156.557655	Typical DTLB conditions
2	CALM	CL	71.312020	−156.609585	Large DTLBs; historical ALT observations
3	Central Plain	CP	71.273177	−156.634896	Typical undisturbed tundra
4	Upper Nunavak Bay	UNB	71.263803	−156.820302	Saturated and unsaturated soils

## 2 Methods

### 2.1 Setup of the GPR unit

**Figure 2:**
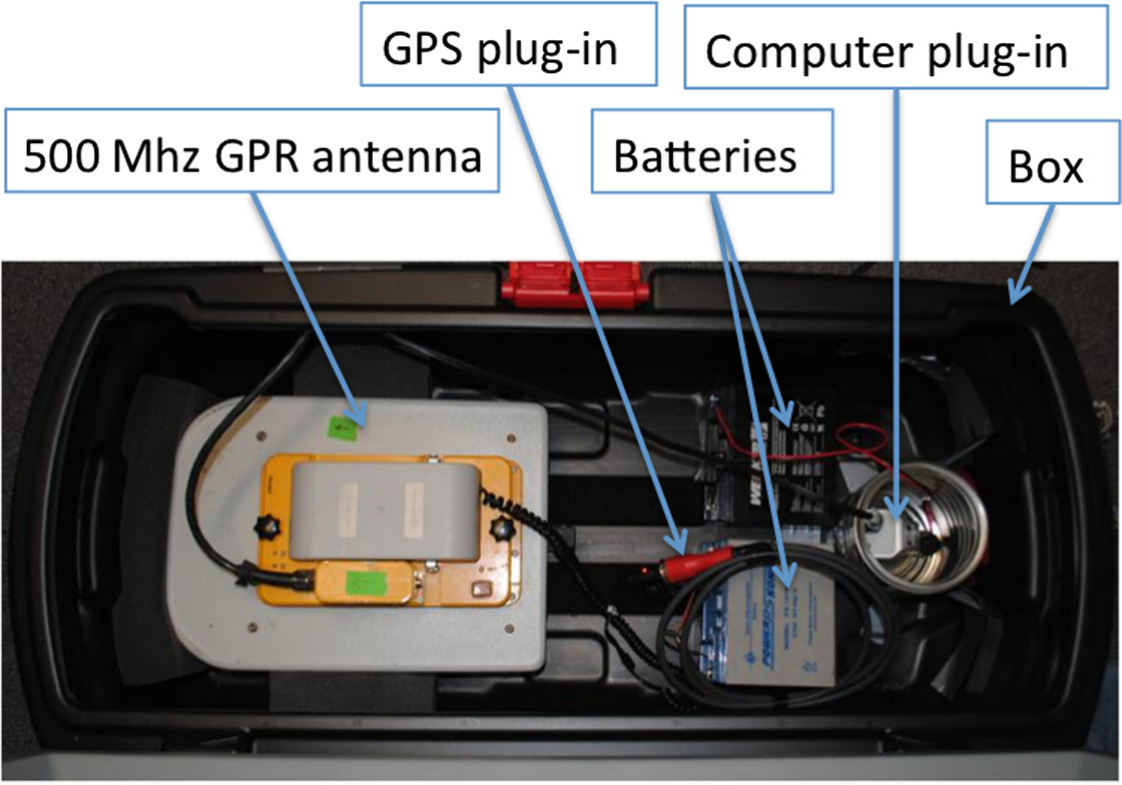
A photo of the ground penetrating radar (GPR) unit setup.

We mounted a Malå 500 MHz shielded antenna in a box to protect it from contact with water and to increase stability of the antenna. During initial testing, we found that the relatively narrow 500 MHz antenna could easily overturn while traversing the hummocky terrain commonly encountered on tundra, and therefore developed the mounting solution within the wider box as shown in Figure 2. We mounted a Garmin GPS 18 on top of the box to record the location of the GPR. The Mala ProEx GPR control unit was carried alongside in a backpack.

### 2.2 GPR data processing

To process the collected data, we did not use any data filters since the reflections at the bottom of the active layer were clearly visible in the raw data. The only signal processing was a ‘dewow’, i.e. subtract mean correction to remove low‐frequency noise. We used the standard time‐zero correction by setting the position of the first arrival as time‐zero for each trace. We manually digitized the radar reflections, known as *picking*, as a quality control measure to verify the signals and reduce interpretation errors due to spurious reflections. More insight on the GPR setup and signal processing are given in Chen *et al*. (2016).

The GPR traces represent ALT values for a footprint of less than 0.15 m^2^, based on the antenna frequency and physical properties of the active layer. We used mechanical probing roughly every 10 min of walking time to calibrate the GPR wave velocity used to convert the two‐way travel time to ALT. For each calibration location, we took the average of three probe measurements adjacent to the GPR antenna. We estimated uncertainty in GPR ALT due to soil moisture variability by propagating the standard deviation of wave velocity through the calculation of ALT from the transit time. Here, we consider soil moisture as a major factor responsible for the variations in velocity. We recognize that soil type and the amount segregated ice within the active layer could also contribute to the variations in velocity.

**Figure 3:**
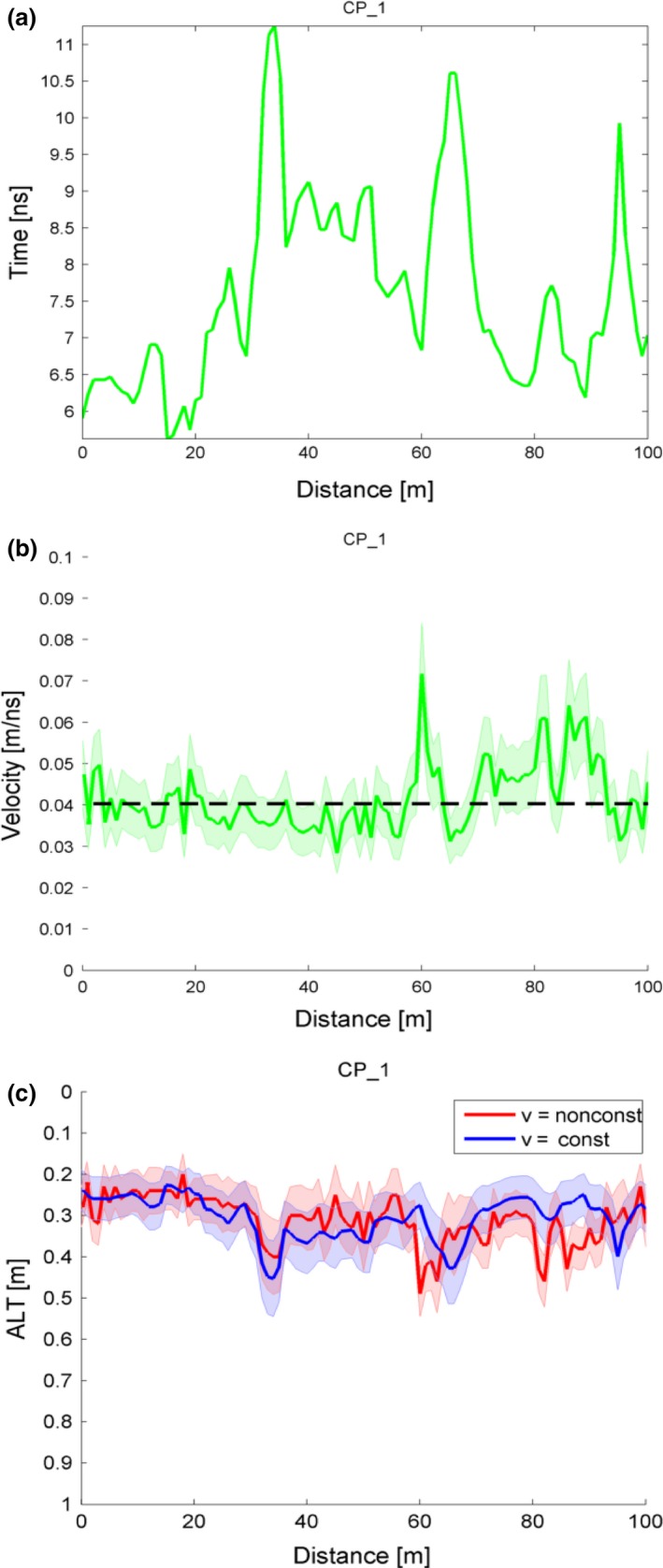
The ground penetrating radar signal processing workflow: (a) one‐way travel time, (b) velocity calculated from probed active layer thickness (ALT) and one‐way travel time, (c) ALT calculated using constant and non‐constant velocities.

The post‐processing workflow is shown in Figure 3: (1) Figure 3(a) shows extracted one‐way travel time (from transmitter to permafrost table) in nanoseconds; (2) Figure 3(b) shows estimated velocity in green with shaded uncertainty in grey and mean velocity in dash‐black line; (3) Figure 3(c) shows estimated ALT using mean and varying velocity.

Table 2 lists the average velocity values for each site, the standard deviation (SD) of the velocities and the depth uncertainty. All depths at each site are estimated using a single velocity and have a single associated depth uncertainty. To calibrate the GPR wave velocity, we used available mechanical probing data, which were used to convert the travel time to velocity. Velocity was calculated by finding GPR points within 1 m of each probe location and using least squares regression to find the slope that minimizes the misfit between the one‐way travel time and probe‐depth. We call the 100 m survey measurements the high‐density (HD) surveys; in the dataset, there are marked as HD (Jafarov *et al*., 2016). For each HD survey, we laid down a 100‐m survey line along the GPR track and used a mechanical probe to measure ALT every metre. Any data points that cannot be paired within 1 m (i.e. the distance between probe locations in HD surveys) were not used in the calibration. The SD velocity was found by calculating the velocity at each probe point using simple time‐depth conversion. The standard deviation of the resulting ensemble was then calculated.

**Table 2:** Averaged velocity statics for four sites in Barrow

Site name	Velocity (m/ns)	SD vel (m/ns)	% vel unc	RMSE depth (± m)
CP	0.041	0.007	16	0.06
BS	0.036	0.005	13	0.05
CL	0.042	0.009	20	0.07
UNB^a^	0.038	0.010	25	0.06

a Due to positioning errors with the GPR‐GPS, only the velocity is calibrated for this site. All other statistics are assumed to be averages similar to the other sites.

We summarize each HD survey in Table 3. We used these HD probe measurements to calibrate the average wave velocity and standard deviations at each study site. All the ALT data measured by using mechanical probes are included in the dataset. The UNB site is marked red in Table 3 due to failure of the GPR data collection at two corresponding survey transects and it is not available in the HD dataset.

**Table 3:** Active layer thickness mechanical probe survey time and locations of the start and end points. The UNB survey marked in red because we are missing the high‐density data measured by GPR

Number	Code^a^	Date	Start	Latitude (deg)	Longitude (deg)	End	Latitude (deg)	Longitude (deg)
1	UNB_1	8/10/2013	15:45	71.26644444	−156.82070830	16:36	71.26555556	−156.82041670
2	UNB_2	8/10/2013	17:22	71.26275000	−156.80930560	17:45	71.26336111	−156.81108330
3	BS_1	8/11/2013	9:40	71.26152778	−156.61797222	10:27	71.26161111	−156.61533333
4	BS_2	8/11/2013	11:00	71.25977778	−156.59388889	11:38	71.25902778	−156.59338889
5	BS_3	8/11/2013	12:55	71.25708333	−156.56508333	13:23	71.25661111	−156.56283333
6	BS_4	8/11/2013	14:05	71.25566667	−156.55086111	14:36	71.25536111	−156.54836111
7	BS_5	8/12/2013	13:15	71.25583333	−156.55372222	13:37	71.25494444	−156.55313889
8	BS_7	8/12/2013	14:35	71.24638889	−156.55869444	15:11	71.24666667	−156.56113889
9	CP_1	8/12/2013	11:13	71.27336111	−156.63983333	11:58	71.27380556	−156.63794444
10	CL_1	8/12/2013	9:55	71.32127778	−156.61750000	10:26	71.32072222	−156.61952778
11	CL_2	8/14/2013	11:30	71.31127778	−156.63036111	11:52	71.31063889	−156.62900000
12	CL_3	8/14/2013	13:11	71.30433333	−156.61033333	13:36	71.30513889	−156.61147222
13	CL_4	8/14/2013	14:41	71.31650000	−156.61094444	15:12	71.31622222	−156.61347222
14	CL_5	8/15/2013	11:09	71.31492000	−156.59113000	11:32	71.31434000	−156.58940000
15	CL_6	8/15/2013	13:25	71.31026000	−156.58823000	13:46	71.31112000	−156.58922000

a Code: Upper Nunavek River (UNB), Big Spot (BS), Central Plain (CP), and CALM (CL).

The Barrow GPR dataset consist of raw and processed data. Both processed and raw data are organized according to the corresponding site with codes shown in Table 1. The raw data files are provided for GPR experts who wish to draw additional insights by applying their own data processing algorithms.

### 2.3 Estimating volumetric water content

We also calculated volumetric water content (VWC) for all high‐density probe measurements. The VWC model uses the electromagnetic wave propagation velocity in the substrate, which depends on the material properties (i.e. volumetric water content) of the substrate. The velocity is much slower in pure water than it is in air, with geologic materials falling in between. Therefore, if we have a GPR velocity as determined from the time‐depth conversion using probe ALT as the known depth, we can calculate an average velocity and water content for that interval of the active layer. It is important to use a model that is appropriate for the conditions. Here, we used the Engstrom *et al*. (2005) empirical VWC model developed for active layer soils in Barrow. However, there are other empirical VWC models for particular materials (e.g. Topp *et al*., 1980; Parsekian *et al*., 2012) that could also be adapted for the Barrow soil types.

To get the most reliable results, we used only probe locations where a GPR trace was available. We extracted the one‐way travel time from the raw GPR data for the traces where we made probe measurements within ~20 cm of the GPR antenna based on the recorded ‘GPR time’ that allows us to look at the exact trace of interest.

### 2.4 Uncertainties

We assume that most of the uncertainties in the GPR‐derived ALT come from the models themselves, since there is no positioning error (collocated probe points are independent of GPS positioning errors), the probe error is <0.03 m, and the GPR picking error is estimated <2%. We used the Euclidian norm to calculate the distance between ALT probe data and GPR data in the calibrated dataset. We chose the minimum distance between collocated points to be within 0.5 m. At each location with collocated GPR two‐way travel time and mechanical probe ALT measurements, we calculate the wave propagation velocity *v* as

(1) $$v=2D_{\mathrm{probe}}/t_{2w}$$

where *D*_probe_ is the ALT measured by mechanical probing in meters and *t*_2w_ is a two‐way signal travel time (to the frozen layer and back) in nanoseconds. We do not calculate velocities for GPR traces that do not collocate with a probe point. We calculate the mean $\hat{v}$ and standard deviation *σ*_*v*_ of observed wave propagation velocities for collocated points. Assuming that $\hat{v}$ is representative for the specific site we derive the GPR ALT:

(2) $$D_{\mathrm{GPR}}=\hat{v}t_{2w}/2$$

In addition to the calculated velocities and GPR ALT, we calculate VWC of the active layer soil at the collocated points using the Engstrom *et al*. (2005) empirical VWC model developed for active layer soils in Barrow. We select the 90% threshold for the calculated VWC. Everything greater than the assigned threshold is associated with pure water and not included in the calibrated dataset.

We included uncertainty into the estimated GPR ALT values. The uncertainty sources are independent of one another, so the total uncertainty in the GPR ALT values is the wave velocity and time‐average uncertainty added in quadrature. We calculate covariance coefficients (CV) for two‐way travel time and velocities by dividing the corresponding standard deviations over means. We provide the corresponding uncertainty for every measured and derived parameter in the calibrated dataset using Gaussian error propagation (Hughes and Hase, 2010):

$$e_{v}={\text{CV}}_{t_{2w}}\cdot v$$

(3) $$e_{D_{\mathrm{GPR}}}={\text{CV}}_{v}\cdot D_{\mathrm{GPR}}$$

$$e_{\mathrm{WVC}}={\text{CV}}_{v}\cdot \text{VWC}$$

## 3 Results and discussion

### 3.1 HD survey data

**Figure 4:**
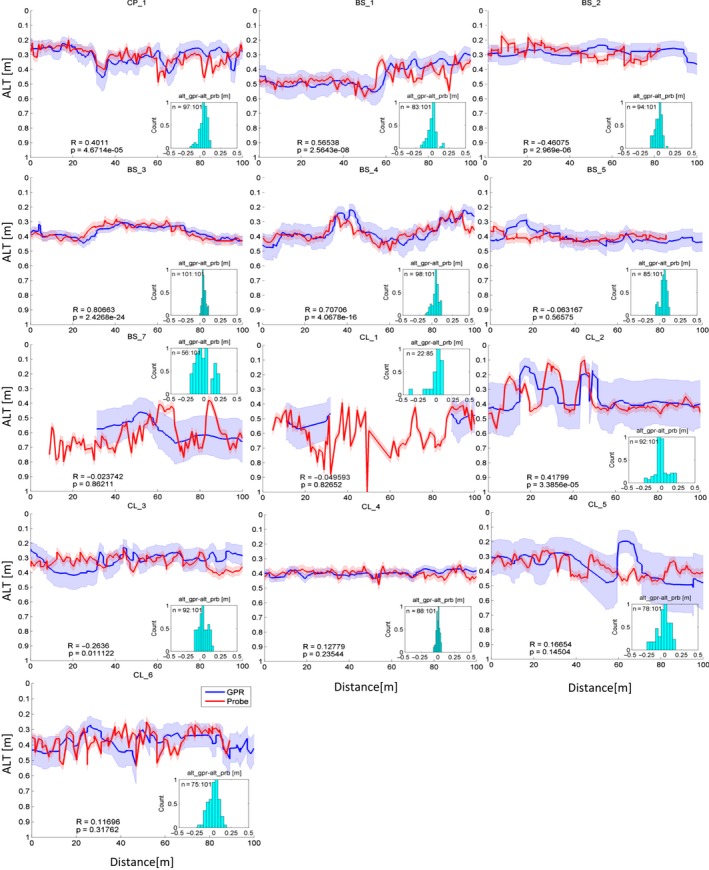
Active layer thickness (ALT) measured with metallic rod (probe data) in red and ALT derived from the ground penetrating radar (GPR) instrument in blue. The red and blue shaded areas stands for the uncertainties in probe and GPR ALTs correspondingly. *R* is a Pearson's correlation coefficient and *p* is a value of significance, *p* < 0.05 stands for the 95% of confidence.

Figure 4 shows the ALT measured using mechanical probing and the estimated ALT using averaged velocity from GPR for 13 HD surveys for sites from Table 2, excluding UNB sites. We did not observe active layer reflections in the GPR data at two HD surveys at the UNB likely due to gravel type soil texture. Each plot in Figure 4 includes the corresponding uncertainties both for GPR and probed ALTs, the error distribution subplots, and correlation coefficient (*R*) with a significance value (*p*). Table 4 summarizes the minimum, mean, and maximum for GPR one‐way travel time and the corresponding velocities along with measured probe ALTs.

**Table 4:** Summaries the overall statistics for the high‐density survey for the GPR and probed ALT

Sites	One‐way travel time (ns)			Velocities (m/ns)			Measured ALT (m)		
	Minimum	Mean	Maximum	Minimum	Mean	Maximum	Minimum	Mean	Maximum
CP_HD_1	5.6259	7.5866	11.2518	0.0283	0.0415	0.0717	0.2	0.3104	0.49
BS_HD_1	7.5673	11.5508	14.3779	0.0249	0.04	0.0574	0.3	0.4546	0.59
BS_HD_2	5.3236	6.3462	7.0124	0.0242	0.0454	0.067	0.17	0.2854	0.38
BS_HD_3	8.828	10.7386	12.9134	0.0285	0.0346	0.0416	0.28	0.3711	0.43
BS_HD_4	5.9839	9.9247	12.953	0.0272	0.0382	0.0637	0.22	0.3705	0.5
BS_HD_5	7.9476	11.3179	13.1211	0.0277	0.0363	0.0516	0.33	0.4056	0.45
BS_HD_7	6.0579	11.8474	13.9269	0.0296	0.0557	0.1304	0.38	0.6303	0.93
CL_HD_1	8.9809	9.69	11.3614	0.0399	0.0612	0.1113	0.39	0.5875	1
CL_HD_2	3.9801	10.2335	12.4922	0.0142	0.0381	0.0895	0.1	0.371	0.57
CL_HD_3	6.2788	8.7821	11.4519	0.0234	0.0388	0.0579	0.24	0.3316	0.42
CL_HD_4	11.041	11.9245	12.9709	0.0293	0.0337	0.0399	0.34	0.4016	0.48
CL_HD_5	4.7152	8.7839	11.805	0.0281	0.0455	0.0997	0.26	0.3745	0.47
CL_HD_6	5.6423	7.7811	11.054	0.0282	0.0487	0.0873	0.25	0.3727	0.54

To complement the direct comparison of the probed *versus* GPR ALTs, plot the distribution of the difference between probed and GPR data and the number of points used in the difference calculation. The total number of probed ALT measurements was 101. Here, we normalized the *y*‐axis by dividing by maximum number of counts in the corresponding histogram. We were not always able to collect probe data due to the roughness of the soil in the gravel‐dominated patches. We defined the overall number of points at each site used in the comparison by the closeness of the two collocated points and availability of the GPR data at the corresponding point.

The CP_1 site shows deeper ALT at along‐track distances between 20 and 40 m, and 60 and 80 m and so on, which illustrates the spatial variation of the ALT in the polygonal tundra. The ALT is deeper in the troughs and shallower inside the polygons. The difference in ALT at along‐track distances greater than 80 m on the CP_1 plot could be attributed to the positioning error between GPR track and probe line. Our observations show that even slight deviation from the GPR track could create a mismatch between the ALT GPR and probe data.

The BS HD surveys might have several positional errors, which contributed to the mismatch between GPR and probe ALTs. Overall, the BS HD surveys show good correspondence between probe and GPR ALT data. The BS_7 HD survey includes gravel patches. We observed higher mixture of gravel in the shoreline of lakes. Typically, when the probed active layer exceeds 50 cm, it corresponds to the soil gravel mixture.

The texture of the soil at the CL_1 site was somewhat similar to the BS_7, mainly gravel patches. Moreover, mechanical probing of the gravel type soil was difficult, which contributed to the large uncertainty of recorded ALT. In addition to complications with using GPR over gravel type soil at CL_1, we also lost some of the GPS signal. The CL_2 site has a range of factors contributing the GPR ALT such as positioning error, gravel patches and velocity averaging.

### 3.2 Volumetric water content

**Figure 5:**
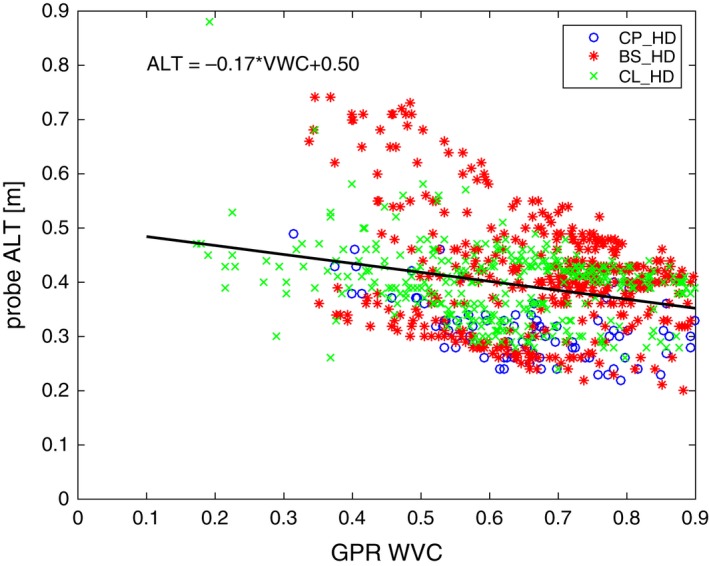
The relationship between probed active layer thickness (ALT) and estimated from the ground penetrating radar (GPR) velocity data volumetric water content (VWC) for Barrow, Alaska. A circle stands for Central Plan (CP) site, a star stands for the Big Spot (BS) site, and a cross stands for the CALM (CL) site correspondingly. The linear fit was calculated for all data points.

Figure 5 shows ALT *versus* VWC for all HD plots shown on Figure 4. Recall that each of these VWCs is calculated based on coincident GPR and probe data, so the velocities and resulting water contents are not dependent on averaging. Figure 5 indicates a weak negative correlation suggesting that active layer is thinner when soils are more saturated with water and thicker for drier soils. Saturated soils require more energy (and longer time) to change from frozen phase to thawed phase in the summer. Similarly, drier soils should have higher ALT and require less time to thaw out. Drier troughs in between ice wedges have deeper ALT, as opposed to the wetter troughs with shallower active layer. However, the standing water in between the polygons could cause deeper active layers than in dry troughs due to heat conducting properties of water. Our result shows that on average the ALT in Barrow varies mainly between 40 and 50 cm.

## 4 Conclusions

The GPR sensing method is a useful technique that improves our understanding of the permafrost distribution in the horizontal and vertical dimensions. The ability of GPR to collect meaningful data depends on the soil type. We found that the 500 MHz GPR instrument was able to capture ALT well in silty and saturated soils but failed to retrieve the correct ALT along the lakeshores with gravel‐dominated soil. It is important to note that probing of the ALT in the gravel‐dominated soil is also challenging, with large uncertainties. Special care must be taken prior to data collection during GPR survey planning. It is important to have enough probe data in order to validate the GPR ALT. In particular, the VWC estimates require corresponding probe data. The GPR‐derived VWC and ALT can be used for airborne derived and model results validation.

## Appendix A

### A.1

Expanded description of the header of the high‐density and processed GPR datasets (see Jafarov *et al*., 2016):

**Table:**

site_ID	site identification code
lat_prb	latitude of the probe data (decimal degrees)
lon_prb	longitude of the probe data (decimal degrees)
alt_prb	active layer thickness measured using mechanical probing (m)
unc_alt_prb	uncertainty associated with the ALT measured using mechanical probing (m)
lat_gpr	latitude of the GPR data (decimal degrees)
lon_gpr	longitude of the GPR data (decimal degrees)
owtt	one‐way travel time (nanoseconds)
velocity	velocity of the signal wave (m/nanoseconds)
unc_vel	uncertainty of the velocity of the signal wave (m/nanoseconds)
cv_vel	covariance coefficient of velocity (m/nanoseconds)
alt_gpr	active layer thickness measured using GPR (m)
unc_alt_gpr	uncertainty associated with the ALT measured using GPR (m)
vwc	volumetric water content (fraction of 1, dimensionless)
unc_vwc	uncertainty of the volumetric water content (dimensionless)
